# Molecular and histopathological detection of *Hepatozoon canis* in red foxes (*Vulpes vulpes*) from Portugal

**DOI:** 10.1186/1756-3305-7-113

**Published:** 2014-03-24

**Authors:** Luís Cardoso, Helder CE Cortes, Osnat Eyal, Antónia Reis, Ana Patrícia Lopes, Maria João Vila-Viçosa, Paula A Rodrigues, Gad Baneth

**Affiliations:** 1 Department of Veterinary Sciences, School of Agrarian and Veterinary Sciences, University of Trás-os-Montes e Alto Douro (UTAD), Vila Real, Portugal; 2 Parasite Disease Group, Instituto de Biologia Molecular e Celular, Universidade do Porto, Oporto, Portugal; 3 Victor Caeiro Laboratory of Parasitology, Instituto de Ciências Agrárias e Ambientais Mediterrânicas, Universidade de Évora, Évora, Portugal; 4 Koret School of Veterinary Medicine, The Hebrew University of Jerusalem, Rehovot, Israel; 5 Animal and Veterinary Research Centre, UTAD, Vila Real, Portugal

**Keywords:** Canine vector-borne diseases, *Hepatozoon canis*, Histopathology, Polymerase chain reaction, Portugal, Red fox, *Vulpes vulpes*

## Abstract

**Background:**

*Hepatozoon canis* is a protozoan tick-borne pathogen of dogs and wild canids. *Hepatozoon* spp. have been reported to infect foxes in different continents and recent studies have mostly used the polymerase chain reaction (PCR) for the detection and characterization of the infecting species. Surveying red foxes (*Vulpes vulpes*) may contribute to better understanding the epidemiology of canine vector-borne diseases, including hepatozoonosis caused by *H. canis* in domestic dogs. The present study investigated the prevalence of *Hepatozoon* spp. by means of histopathology and molecular analysis of different tissues in red foxes from different parts of Portugal.

**Methods:**

Blood and tissues including bone marrow, heart, hind leg muscle, jejunum, kidney, liver, lung, popliteal or axillary lymph nodes, spleen and/or tongue were collected from 91 red foxes from eight districts in northern, central and southern Portugal. Tissues were formalin-fixed, paraffin-embedded, cut and stained with hematoxylin and eosin. Polymerase chain reaction (PCR) amplified a ~650 bp fragment of the 18S rRNA gene of *Hepatozoon* spp. and the DNA products were sequenced.

**Results:**

*Hepatozoon canis* was detected in 68 out of 90 foxes (75.6%) from all the sampled areas by PCR and sequencing. Histopathology revealed *H. canis* meronts similar in shape to those found in dogs in the bone marrow of 11 (23.4%) and in the spleen of two (4.3%) out of 47 foxes (*p* = 0.007). All the 11 foxes found positive by histopathology were also positive by PCR of bone marrow and/or blood. Positivity by PCR (83.0%) was significantly higher (*p* < 0.001) than by histopathological examination (23.4%) in paired bone marrow samples from the same 47 foxes. Sequences of the 18S rRNA gene of *H. canis* were 98–99% identical to those in GenBank.

**Conclusions:**

*Hepatozoon canis* was found to be highly prevalent in red fox populations from northern, central and southern Portugal. Detection of the parasite by histopathology was significantly less sensitive than by PCR. Red foxes are a presumptive reservoir of *H. canis* infection for domestic dogs.

## Background

Hepatozoonosis caused by *Hepatozoon canis* is a canine vector-borne disease (CVBD) reported from the Old World and more recently also from South and North America [1]. The apicomplexan parasite *H. canis* (suborder Adeleorina: family Hepatozoidae) has a life cycle which involves vertebrates as intermediate hosts, including domestic dogs and wild canids, and ticks as definitive hosts [2].

Autochthonous infections with *H. canis* are prevalent in dogs from areas with tropical, subtropical or temperate climates, where their primary vector is the three-host tick species *Rhipicephalus sanguineus*, the brown dog tick [3,4]. Transmission of *H. canis* to the vertebrate hosts typically takes place by ingestion of the arthropod vector containing mature protozoal oocysts with infective sporozoites [5,6]. Nevertheless, intrauterine transmission from a dam to its pups has also been observed in *H. canis* infection [7]. No transfer during the vector blood feeding has been demonstrated for any *Hepatozoon* spp., including *H. canis*, as opposed to most vector-borne pathogens, which are transmitted by their invertebrate hosts via the salivary glands during a blood meal [2]. After ingestion of infected ticks, sporozoites invade mononuclear cells and disseminate via blood or lymph to different visceral organs of the vertebrate hosts, such as the bone marrow, spleen and lymph nodes, where they develop into the meront stage [1]. Merozoites infect leukocytes where the gamont stage of the protozoan, infective for the tick, is found later [8].

In dogs, infection with *H. canis* is often subclinical but may manifest as a mild to debilitating and even life-threatening disease with cachexia, lethargy and anemia [2,9]. *Hepatozoon canis* is detectable by microscopic observation of circulating intracellular gamonts in stained blood smears [10]. The parasite can also be visualized in histopathological specimens, with micromerozoites aligned in a circle around a central opaque core forming the typical “wheel spoke”-pattern mature meront [6]. Molecular techniques, including the polymerase chain reaction (PCR) and real-time PCR are available for sensitive detection, genetic characterization and epidemiological studies of *Hepatozoon* spp. infections in blood and other tissues [11-15].

Red foxes (*Vulpes vulpes*) have been demonstrated to be infected with *Hepatozoon* spp. morphologically resembling *H. canis*[16] and as seropositive for the parasite [17] in areas where it is prevalent in domestic dogs. Moreover, *H. canis* infections were molecularly confirmed in red foxes in Spain [18], Slovakia [19], Croatia [20] and Italy [21]. Infections with *Hepatozoon* spp. other than *H. canis* have been reported in other species of foxes including the gray fox (*Urocyon cinereoargenteus*), the crab-eating fox (*Cerdocyon thous*) and the wild Pampas fox (*Lycalopex gymnocercus*) [22-24]. Surveying foxes, as hosts that share habitats with dogs, may contribute to a better understanding of the epidemiology of CVBD such as hepatozoonosis due to *H. canis*. The present study aimed at investigating the prevalence of *Hepatozoon* spp. by means of histopathology and molecular analysis in red foxes from different regions of Portugal.

## Methods

Eighty-eight corpses of wild red foxes (*V. vulpes*) shot during the official hunting season or killed in road accidents were obtained between November 2008 and March 2010. These animals originated from the districts of Viana do Castelo (n = 9), Bragança (n = 13), Vila Real (n = 20), Braga (n = 3) and Porto (n = 2), in the north; Aveiro (n = 2), in the centre; and Évora (n = 39), in the south of Portugal [25]. Two additional red foxes from the southern district of Setúbal and another one from Bragança were presented alive to the Center for Wildlife Rehabilitation, UTAD Veterinary Hospital (UTAD-VH).

The corpses were refrigerated (at 4°C) for no more than 72 h or kept frozen (at −20°C) and thawed before collecting the samples. During necropsy, clotted blood was collected from the right atrium or chest cavity and bone marrow from the femoral bone, with sterile equipment, and stored at −20°C until further processing. Blood from the jugular or cephalic veins of the three living foxes was collected into EDTA tubes and also kept under the same freezing conditions as above. This study was approved by the UTAD-VH ethical committee as complying with the Portuguese legislation for the protection of animals (Law no. 92/1995).

Out of 52 foxes whose gender was observed, 23 were females and 29 males; gender was not recorded for 39 of the 91 foxes. Age was determined by morphologic characteristics and tooth eruption pattern and wear [26] in 48 foxes and ranged from 1.0 to 7.5 years, with a median value of 2.5 years (interquartile range: 1.5–3.5). Fourteen foxes were classified as juveniles (less than 2 years of age) and 34 as adults (2 years or more) [27].

Samples of heart, hind leg muscle (biceps femoris), jejunum, kidney, liver, lung, popliteal or axillary lymph nodes, spleen and tongue were further collected from 47 foxes of northern Portugal. For routine histopathological analysis, tissues were formalin-fixed, paraffin-embedded, cut at 3 μm thickness and stained with hematoxylin and eosin (H&E).

DNA was extracted from blood, bone marrow, heart, hind leg muscle, liver, lung, popliteal lymph node and spleen samples with commercial purification kits (QIAamp® DNA Blood Mini or QIAamp® DNA Mini; Qiagen, Valencia, CA, USA), according to the manufacturer’s instructions. Polymerase chain reaction (PCR), using primers (125 nM each) Hep-F (5′-ATA CAT GAG CAA AAT CTC AAC-3′) and Hep-R (5′-CTT ATT ATT CCA TGC TGC AG-3′), amplified an approximately 650 bp partial sequence of the 18S rRNA gene of *Hepatozoon* spp. [15,28]. The following conditions were used for amplification: 95°C for 5 min; 35 cycles of 95°C for 20s, 57°C for 30s, and 72°C for 90 s; and 72°C for 5 min. PCR was performed using Syntezza PCR-Ready High Specificity (Syntezza Bioscience, Jerusalem, Israel), with positive (*H. canis*) and negative control samples run with each reaction. PCR products were sequenced using the BigDye Terminator v3.1 Cycle Sequencing Kit and an ABI PRISM 3100 Genetic Analyzer (Applied Biosystems, Foster City, CA, USA), at the Center for Genomic Technologies, Hebrew University of Jerusalem, Israel. DNA sequences were evaluated with the ChromasPro software version 1.33 and compared for similarity to sequences available from GenBank, using the BLAST 2.2.9 program (http://www.ncbi.nlm.nih.gov/BLAST/).

For statistical analysis, the Chi-squared or Fisher’s exact tests compared proportions of positivity. The McNemar’s test was used to compare results obtained from paired samples (i.e., from the same animal). A *p* value <0.05 was considered as statistically significant [29]. The exact binomial test estimated confidence intervals (CI) for proportions, with a 95% confidence level. Analyses were done using StatLib or SPSS 11.5 software for Windows.

## Results

**Figure 1:**
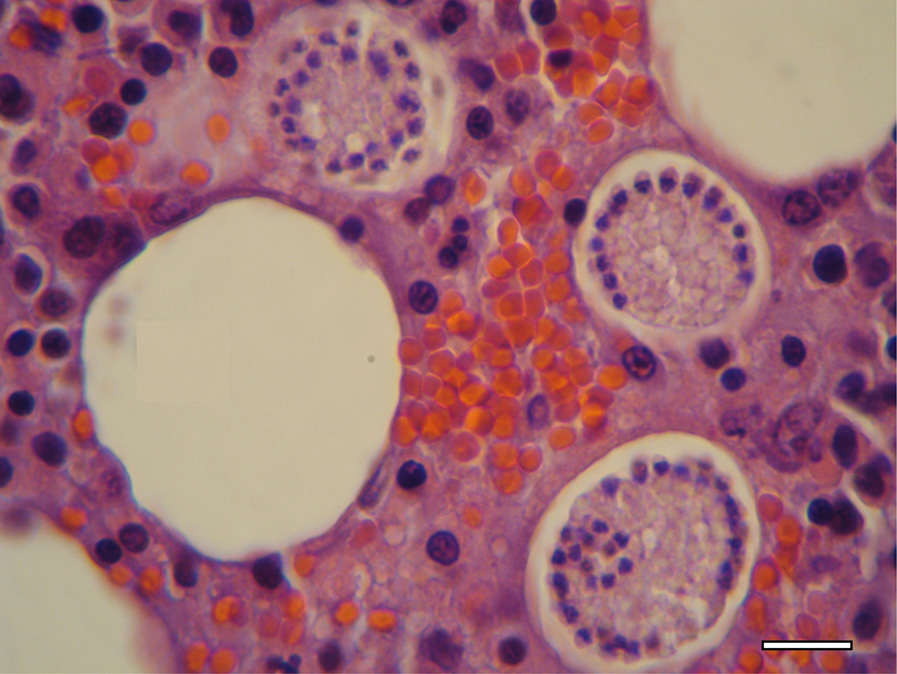
**Bone marrow histopathological specimen from a red fox.** Three mature meronts of *H. canis* with a typical wheel-spoke structure, containing micromerozoites aligned in a circle around a central opaque core (H&E, scale-bar = 20 μm).

**Figure 2:**
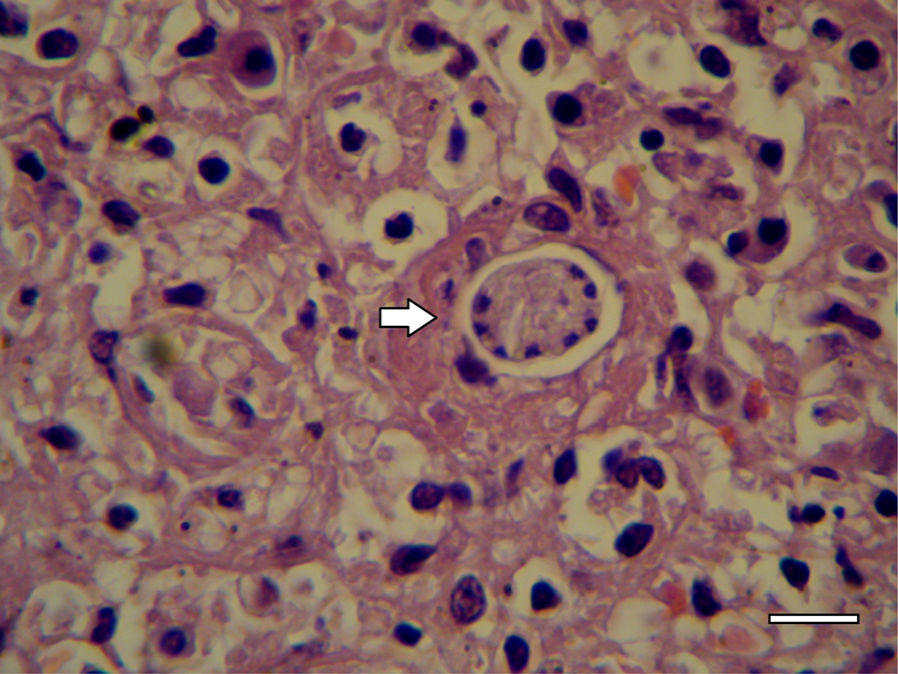
**Spleen histopathological specimen from a red fox.** A developing meront of *H. canis* (arrow) (H&E, scale-bar = 20 μm).

Histopathology revealed *Hepatozoon* meronts with wheel spoke structure (Figure 1) in the bone marrow of 11 (23.4%) out of the 47 examined foxes from the north of Portugal. Meronts were also found in the spleen (Figure 2) of two of those 11 positive foxes, thus representing 4.3% out of the 47 foxes (*p* = 0.007). No *Hepatozoon* developmental forms were detected in the remaining histopathological samples from other tissues (heart, hind leg muscle, jejunum, kidney, liver, lung, popliteal or axillary lymph nodes and tongue). No other histological abnormalities were observed in any of the tissues histologically examined. However, some samples showed autolytic alterations, in spite of the attempts to minimize postmortem effects.

By PCR and sequencing (Table 1), *H. canis* was detected in 68 out of 90 foxes (75.6%; 95% CI: 64.5–83.2%), from all the eight sampled districts. *Hepatozoon canis* was identified in 40 (81.6%) out of 49 foxes from the northern/central districts and in 28 (68.3%) out of the 41 foxes from the southern districts (Table 1). Due to discrepancy in the number of paired DNA samples between north/centre and south, statistical comparison of blood and bone marrow combined results was not performed by geographical region. In fact, 50 blood and 47 paired bone marrow samples were available from the 50 northern/central foxes, whereas only two paired blood samples were tested for 32 bone marrow samples among the 41 southern foxes.

**Table 1:** **Results of PCR for *Hepatozoon *** **spp. and sequencing from blood and bone marrow DNA samples of red foxes from Portugal**

**Region in Portugal**	**No. of positive foxes/no. of foxes tested (%)**			
	**PCR – blood**	**PCR – bone marrow**	**PCR – blood and/or bone marrow**	**PCR and sequencing identifying *H. canis *– blood and/or bone marrow**
North and centre	37/50 (74.0)	39/47 (83.0)	41/50 (82.0)	40/49 (81.6)
South	6/11 (54.5)	23/32 (71.9)	29/41 (70.7)	28/41 (68.3)
Total	43/61 (70.5)	62/79 (78.5)	70/91 (76.9)	68/90 (75.6)

PCR for *Hepatozoon* spp. was positive in 36 (73.5%) of 49 DNA blood samples and 39 (79.6%) of 49 paired bone marrow samples, with no statistically significant difference between positivity in the blood and bone marrow (*p* = 0.375). Based on blood and bone marrow combined sequencing results, 19 (82.6%) female and 23 (82.1%) male foxes were positive for *H. canis*. There were 10 (71.4%) juvenile and 28 (84.8%) adults positive for *H. canis*, also based on combined sequencing results from blood and bone marrow. Differences were not statistically significant between genders (*p* = 0.419) or age groups (*p* = 1.0).

Positivity by PCR (83.0%) was significantly higher (*p* < 0.001) than by histopathological examination (23.4%) in paired bone marrow samples from the same 47 foxes. All the 11 foxes found positive by histopathology were also positive by PCR of bone marrow and/or blood. The presence of *H. canis* in multiple different tissues was assessed by PCR in a subset of six foxes positive by histopathology of bone marrow and in six foxes negative by histopathology but positive by PCR in blood. No statistically significant differences were found between the two subsets by comparing the proportion of positive PCR results between histopathology-positive and -negative foxes for the same tissues (Table 2).

**Table 2:** ***Hepatozoon *** **spp. PCR results in eight tissues of foxes found with (positive) or without (negative) wheel-spoke meronts by histopathological examination of bone marrow**

**Histopathology**	**PCR (no. of positive samples/no. of samples tested)**							
	**Blood**	**Bone marrow**	**Heart**	**Hind leg muscle**	**Liver**	**Lung**	**Lymph node**	**Spleen**
Positive (n = 6)	5/6	6/6	6/6	4/6	6/6	6/6	6/6	6/6
Negative (n = 6)	6/6*	6/6	4/6	3/6	5/6	6/6	5/6	5/6

* Inclusion of histopathologically negative foxes was based on a positive blood PCR result.

Of the 18S rRNA DNA sequences obtained by sequencing of the PCR product and searching for the closest GenBank match, 40 sequences from the northern/central red foxes were 98–99% identical to GenBank GU376457 (*H. canis* from an Italian red fox [21]). Of the sequences from the southern foxes, 22 were 99% identical to GU376457, four were 99% identical to EU289222 (*H. canis* from a Taiwanese dog), one was 99% identical to AY150067 (*H. canis* from a Spanish red fox [30]) and another was 99% identical to FJ608736 (*H. canis* from an Italian dog).

## Discussion

A high prevalence of *H. canis* infection in foxes has been found by PCR in surveys from several countries in southern Europe. By blood PCR the prevalence of *H. canis* in red foxes from northern Spain (28.6% [2/7]) [31] was considerably lower than that found in the blood of foxes from Portugal in this study (70.5%). By spleen PCR, the prevalence levels of *H. canis* in red foxes were 90% (18/20) in central Spain [18], 23.0% (44/191) in Croatia [20] and 16.4% (16/119) in Italy [21]. In a previous study from southern Portugal, 143 (48%) out of 301 red foxes assessed by microscopy of blood, bone marrow and impression smears of spleen and lymph node, were found infected with meronts or gamonts [16]. In Israel, by means of an enzyme-linked immunosorbent assay, 20 (23.8%) out of 84 fox sera tested were positive for antibodies to *H. canis*[17]. The diversity of results observed among studies may represent distinct prevalence levels by geographical localization, but may also derive from different techniques used, such as serology indicating exposure and PCR detecting infection, or different tissues sampled [32].

The presence of *H. canis* in striated muscle has been a point of debate as it was detected by PCR in the heart and striated muscle tissues of red foxes from Slovakia [19]. Although *Hepatozoon americanum*, a species found in North America, primarily infects skeletal and cardiac muscles [33], this is not the case for *H. canis*, which mostly infects hemolymphoid tissues of domestic dogs [2]. In the present study, spleen, heart and hind leg muscle samples from 12 foxes were selected for PCR based on previous histopathological or blood-PCR positivity. Our study also indicated that fox muscle tissues were positive by PCR for *H. canis*, but no histopathological evidence for infection of muscle cells was found in the 12 foxes from which multiple tissues were studied. The lack of histopathological evidence for infection of fox muscles by *H. canis*, yet the positive PCR of these tissues, could be due to a low level of true infection in the muscle itself missed by histopathology, or more likely to trapping of parasite from blood or hemolymphoid tissue in the sampled muscles. The autolytic process could have also contributed to a lower sensitivity of the histopathological analysis. In the present study, *Hepatozoon* spp. meronts were found by histopathology only in bone marrow (Figure 1) and spleen (Figure 2), with the former yielding a higher level of detection than any other tissue. This finding reinforces the idea that merogony occurs mainly in the bone marrow of red foxes, as previously suggested [16]. In addition, the present study represents the first comparison of histopathology and PCR for the detection of *H. canis* in red foxes, with the latter demonstrated to be significantly more sensitive for that purpose.

The vector of *H. canis*, the three-host tick *R. sanguineus*, has been found to infest mammalian hosts from all the 18 districts of continental Portugal [34]. The transmission of *H. canis* in ticks may occur transstadially from larvae to nymphs [35] and from these to the adult stages of *R. sanguineus*[8]. Both nymphs and adults containing *H. canis* sporozoites within oocysts can infect canids upon ingestion of ticks. Transovarial transmission, i.e. from female ticks to eggs, has not been demonstrated for *H. canis*[35]. Vertical intrauterine transmission of *H. canis*[7] might also contribute to the spread of this infection and explain its high prevalence in foxes. The fact that there was no difference between the prevalence of infection in juvenile and adult foxes might indicate that foxes are infected at a young age, by a vector or by transplacental transmission. Another mode of transmission not yet demonstrated for *H. canis*, but that could be associated with foxes, is predation by a canid host on another intermediate host, as shown for *H. americanum*[36].

In general, the high prevalence levels of *Hepatozoon* spp. found in red foxes contrasts with the much lower prevalence levels in dogs from the same geographical areas [16,21,37]. Out of 331 dogs from kennels and shelters in southern Portugal, 21.1% were found to be positive for *H. canis* by PCR in blood [38]. Another recent investigation that molecularly assessed 320 cats from northern and central Portugal did not reveal any cat PCR-positive for *H. canis*, but 15.6% of the animals were PCR-positive for *Hepatozoon felis*[39]. *Hepatozoon canis* is orally transmitted by ingestion of infected ticks, and foxes are probably more likely than dogs or cats to accidentally ingest arthropods, by feeding more often on live prey or carrion [30]. With such a high prevalence of infection as shown in the present study in Portugal, foxes are likely to be a major reservoir of *H. canis* for domestic dogs. Interestingly, foxes existed in the world long before there were domestic dogs [40] and could be speculated to have been the original source of *H. canis* to other wild and domestic canids.

Some of the partial *H. canis* 18S rRNA gene sequences obtained from foxes in the present study are most similar to those previously identified in Spanish and Italian red foxes (GenBank AY150067 and GU376457, respectively). However, *H. canis* sequences found in European foxes are also very similar to those found in dogs in the same countries. Therefore, the epizootiological importance of possible fox *H. canis* strains needs to be studied further and with longer DNA sequences before conclusions on such strains can be deduced [20,32].

## Conclusions

In conclusion, *H. canis* has been demonstrated to be highly prevalent in red fox populations from northern/central and southern Portugal. Detection of *H. canis* by histopathology was shown to be significantly less sensitive than PCR. The high prevalence of *H. canis* in red foxes indicates that they are likely to be a major reservoir for infection of domestic dogs.

## Competing interests

The authors declare that they have no competing interests.

## Authors’ contributions

LC designed the study, collected samples, analysed data and wrote the manuscript; HCEC designed the study, collected samples and assisted in drafting the manuscript; OE performed PCR and sequencing, and analysed data; AR, APL and MJV-V collected samples; PAR collected samples, performed histopathology and analysed data; GB designed the study, performed PCR and sequencing, analysed data and revised the manuscript. All authors read and approved the final version of the manuscript.
